# Combined Use of Aspirin and Statins Is Associated With Increased Risk of Metabolic Dysfunction–Associated Steatotic Liver Disease: A Study From National Health and Nutrition Examination Survey

**DOI:** 10.1155/grp/9931208

**Published:** 2025-10-05

**Authors:** Zheng-Wu Jiang, Ya-Si Wang, Wen-Liang Tan, Bin Feng, Ming-Chang Zhang, Han-Qi Liao, Jian Wan, Xun Chen, Zhi-Qin Xie

**Affiliations:** ^1^Department of Hepatobiliary and Pancreatic Surgery, Medical Center of Digestive Disease, Zhuzhou Hospital Affiliated to Xiangya School of Medicine, Central South University, Zhuzhou City, Hunan Province, China; ^2^Second Department/The Second Ward, Third Social Welfare Institute of Changsha City, Changsha, Hunan, China; ^3^Department of Hepatobiliary Surgery, Hunan Provincial People's Hospital (The First Affiliated Hospital of Hunan Normal University), Changsha, China; ^4^Hunan Key Laboratory for the Prevention and Treatment of Biliary Tract Diseases, Changsha, China

**Keywords:** advanced liver fibrosis, aspirin, association, metabolic dysfunction–associated steatotic liver disease, statins

## Abstract

**Background and Aim:**

Aspirin and statins are often taken together in clinical practice. The impact of combined use of aspirin and statin (CAS) therapy on individuals with metabolic dysfunction–associated steatotic liver disease (MASLD) or advanced liver fibrosis (LF) remains uncertain. This study is aimed at exploring their associations in US adults.

**Methods:**

This cross-sectional study used data from the 2011 to 2018 National Health and Nutrition Examination Survey, and 5889 participants were included. MASLD is based on the 2023 international expert consensus definition. Advanced LF is defined based on three commonly-used noninvasive fibrosis scores. Furthermore, prescriptions of aspirin and statins were categorized into untreated, single, and CAS to assess their potential associations with MASLD or advanced LF.

**Results:**

The CAS group consisted mostly of older (66 years), male (58.5%), non-Hispanic White (74.5%) individuals who were highly educated (58.0%), obese (45.7%), never smokers (43.8%), moderate drinkers (38.5%), and had hypertension (75.3%) and diabetes (39.9%). CAS was associated with a 49% higher risk of MASLD (OR: 1.49; 95% CI: 1.12–1.99) and a 40% higher risk of advanced LF (OR: 1.40; 95% CI: 1.02–1.92) after full adjustment. Notably, stratification and interaction analyses demonstrated that metabolic parameters such as overweight/obese, diabetes, and hypertriglyceridemia significantly influenced the association between CAS and MASLD or advanced LF.

**Conclusions:**

In summary, the study indicates a positive correlation between CAS and MASLD or advanced LF. Using CAS should be cautious to avoid the increased risk of the above diseases.

## 1. Introduction

Metabolic dysfunction–associated fatty liver disease (MAFLD), previously referred to as nonalcoholic fatty liver disease (NAFLD) [1], is a pathological condition characterized by dysregulated lipid metabolism, which leads to hepatic steatosis and may progress to liver fibrosis, cirrhosis, and malignancy [2]. Currently, more than a quarter of the global population is affected by MAFLD, positioning it as a predominant cause of chronic liver disease [3]. The rising prevalence of obesity and diabetes has significantly contributed to the increasing incidence of MAFLD, with rates escalating from 22% to 36% over recent decades [4]. This persistent condition is anticipated to continue into the future, resulting in an escalating economic burden and increased pressure on public health systems.

According to the Delphi consensus, NAFLD and MAFLD have been redefined as metabolic dysfunction–associated steatotic liver disease (MASLD) [5]. Unlike the stringent and complex criteria for MAFLD, which necessitate the presence of Type 2 diabetes (T2DM), overweight/obesity, or at least two additional metabolic variables, MASLD simplifies the diagnostic criteria by requiring only one of five metabolic risk factors [5]. Consequently, MASLD can identify approximately 1.5% more individuals than MAFLD [6]. This condition is expected to persist, thereby perpetually exacerbating the economic burden and imposing stress on public health systems. Currently, there is no pharmacotherapy available for either MAFLD or MASLD; the recommended management strategy involves lifestyle modifications aimed at addressing metabolic disorders and mitigating cardiovascular risks [7].

Aspirin and statins are commonly coprescribed to mitigate metabolic abnormalities and reduce cardiovascular risks. Aspirin and statins are frequently coprescribed to address metabolic abnormalities and diminish cardiovascular risks. According to Fuster and Sweeny, over 50 million individuals in the United States use aspirin for the prevention of cardiovascular diseases, with global consumption reaching approximately 40,000 tons annually [8]. Over the past decade, the number of adult statin users in the United States has surged by nearly 80%, rising from 21.8 million in 2002–2003 to 39.2 million in 2012–2013 [9]. Prior research has demonstrated a correlation between regular aspirin use and a decreased prevalence of NAFLD, as well as a reduced risk of fibrosis progression [10, 11]. Numerous studies have indicated that statins are both effective and safe in the treatment of liver inflammation and fibrosis associated with nonalcoholic steatohepatitis (NASH) and NAFLD [12]. Despite statins and aspirin being classified as hepatotoxic agents [13], the impact of their combined use on the progression of MAFLD or MASLD remains largely unexplored.

Consequently, this study employed data from the National Health and Nutrition Examination Survey (NHANES) spanning 2011–2018 to examine the potential benefits or risks associated with the combined use of aspirin and statin (CAS) on the progression of MAFLD, MASLD, or advanced liver fibrosis.

## 2. Methods

### 2.1. Study Population

**Figure 1:**
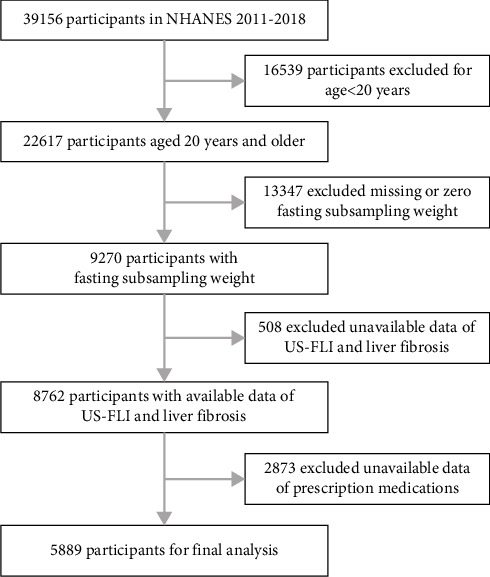
A flowchart showing the selection of study participants. NHANES, National Heathy and Nutrition Examination Survey; US-FLI, US-Fatty Liver Index.

NHANES is a national study designed to obtain an exemplary sample of the noninstitutionalized American residents. Approximately 5000 participants from across the country participate in the survey annually, which has been ongoing since 1999. Data from four NHANES cycles spanning the years 2011–2018 were utilized for the purposes of this study. A total of 39,156 participants completed all surveys in the study. Of these, 16,539 individuals were excluded due to being younger than 20 years old, and an additional 13,347 were further excluded for lacking fasting subsampling weight. Ultimately, the study included 5889 participants after excluding those with incomplete data (Figure 1). Data from the NHANES are publicly accessible and were approved by the NHANES Institutional Review Board (IRB) and the Ethics Review Board (ERB) of the National Center for Health Statistics. Moreover, individual members endorse a familiar consent form [14].

### 2.2. Exposure: Aspirin and Statin Use

Data for evaluating drug usage is sourced from the NHANES public database and is ascertained through data collected from the NHANES 2011–2018 annual questionnaire. Specifically, the utilization of aspirin was assessed through the following three inquiries: “Do you follow your doctor's advice to use low-dose aspirin every day to prevent illnesses such as heart attacks, strokes, and cancer? Do you remember being told to do this?”, “Do you use low-dose aspirin yourself?”, or “Are you currently using low-dose aspirin?”. Utilizing dichotomous questionnaires, we established a binary variable to denote current aspirin usage and inquired about statin usage by asking participants if they had utilized any prescription medications within the previous 30 days. Participants were instructed to present all medication containers if their answer was “yes.” Statin usage was identified through the specific generic names of atorvastatin, simvastatin, pravastatin, rosuvastatin, lovastatin, pitavastatin, fluvastatin, cerivastatin, and combination products.

### 2.3. Outcome: MAFLD, MASLD, and Advanced LF

US-Fatty Liver Index (US-FLI) scores of 30 and above indicate liver steatosis based on prior studies [15]. The expert consensus defines MAFLD as hepatic steatosis diagnosed through histological imaging or blood biochemical markers, in combination with either overweight (body mass index [BMI] ≥ 25 kg/m^2^) or obesity (BMI ≥ 30 kg/m^2^), T2DM, or signs of metabolic dysfunction [16]. Metabolic disorder is delimited by the existence of no fewer than two metabolic risk factors. These risk factors encompass (a) waist circumference (WC) of 102 cm or more in men and 88 cm or more in women; (b) hypertension (HBP) was defined as 130 or 85 mmHg for diastolic pressure, with or without medication; (c) plasma triglyceride (TG) levels of 150 mg/dL or higher or higher with or without medication; (d) plasma high-density lipoprotein cholesterol (HDL-C) below 40 mg/dL in men and below 50 mg/dL in women with or without medication; and (e) prediabetes mellitus (fasting plasma glucose 5.6–6.9 mmol/L, or glycohemoglobin (HbA1c) 5.7%–6.4%). C-reactive protein was not included due to its unavailability in NHANES 2011–2014.

In 2023, experts reached a consensus to establish the diagnostic criteria for MASLD [5]. MASLD is characterized by liver steatosis and the presence of at least one of five cardiovascular metabolic risk factors: (1) BMI ≥ 25 kg/m^2^ or WC > 94/80 cm in men and women, (2) HbA1c ≥ 5.7%, (3) “b” of MAFLD, (4) “c” of MAFLD, and (5) “d” of MAFLD. Advanced LF was evaluated using serological noninvasive fibrosis indices, such as the nonalcoholic fatty liver disease fibrosis score (NFS), fibrosis index based on four factors (FIB-4), and aspartate aminotransferase-to-platelet ratio index (APRI) [17–19]. Participants with elevated APRI (> 1.5), FIB-4 (> 2.67), or NFS (> 0.676) were identified as high endangerment for advanced fibrosis in this study [20].

### 2.4. Covariates

According to published literature and clinical experience, we selected the following variables in this study: gender, age, race/ethnicity, poverty–income ratio (PIR), education level, T2DM, hepatitis B virus (HBV) infection, hepatitis C virus (HCV) infection, HBP, physical activity level, BMI, smoking status, alcohol consumption, alanine aminotransferase (ALT), aspartate aminotransferase (AST), albumin (ALB), gamma glutamyl transferase (GGT), total cholesterol (TC), TGs, platelet count (PLT), and WC.

Upon conducting screening for outliers and missing data in the dependent variables, it was observed that the dataset for participants aged over 40 was absent. Consequently, this study categorized age into two distinct groups: 40–59 and 60 years and above. Additionally, race/ethnicity was categorized as Mexican American, other Hispanic, non-Hispanic White, non-Hispanic Black, and other races. Furthermore, family income was evaluated by dividing it by the PIR and classifying it into three categories: < 1.3, 1.3–1.8, and ≥ 1.8. The educational attainment of participants was ascertained through interviews, categorizing them as having education levels above high school, high school, and below high school. The identification of diabetics based on a self-reported history of diabetes or a HbA1c level of 6.5% or higher was performed [21]. Further, HCV and HBV infections were determined by positive diagnostic tests or self-reports [22, 23].

Additionally, individuals were divided into three categories, namely, active (meeting or surpassing the suggested levels of vigorous activity [≥ 75 min/week] and moderate activity [≥ 150 min/week] [24]), less active (below the recommended activity levels), and inactive (not participating in physical activity). Participants were further stratified based on their BMI as underweight (< 20.0 kg/m^2^), normal weight (20.0–25.0 kg/m^2^), overweight (25.0–29.9 kg/m^2^), and obese (≥ 30.0 kg/m^2^). Smoking status was categorized as never, former (had quit smoking), and current (smoke now and smoke at least 100 cigarettes). The National Institute on Alcohol Abuse and Alcoholism (NIAAA) guidelines classify alcohol consumption as never, moderate (one drink per day for women or one to two drinks per day for men), or heavy (two or more drinks per day for women or three or more drinks per day for men). Furthermore, laboratory tests provided data on ALT, AST, ALB, GGT, TC, TG, and PLT, while body measures yielded WC measurements.

### 2.5. Statistical Analysis

The continuous data was analyzed through weighted linear regression with mean ± standard deviation (SD). Furthermore, using weighted percentages with 95% confidence intervals (95% CIs) for categorical variables, the chi-square test was conducted to assess variances among groups. A multivariable logistic regression analysis was conducted to examine the link between CAS and MAFLD, MASLD, or advanced LF. Model 1 was an unadjusted model, and Model 2 was adjusted for gender, age, and race. Model 3 was further adjusted for education, PIR, smoking status, alcohol use, HBV and HCV infection, ALT level, and physical activity status in addition to Model 2. The final model (Model 4) added BMI, diabetes, and hypertriglyceridemia on the basis of Model 3. Subgroup analyses were conducted to determine the impact of age, gender, race, BMI, hypertriglyceridemia, and diabetes on the study consequences. In Model 4, the potential interaction between the results and subgroup variables is tested by interaction and demonstrated as illustrated in a forest plot.

Data analysis was performed using Stata 17.0 (StataCorp, College Station, Texas, United States) and EmpowerStats (http://www.empowerstats.com, X&Y Solutions, Inc., California, United States). National prevalence rates were determined by adjusting for appropriate fasting weights to accommodate the intricate sampling design utilized in the NHANES. Statistical significance was assessed using two-tailed tests, with a significance level of *p* < 0.05 demonstrating statistical significance.

## 3. Results

### 3.1. Characteristics of Included Participants

This study encompassed 5889 participants from four NHANES cycles, as depicted in Figure 1. Among them, 903 individuals were taking aspirin alone, 707 were taking statins alone, and 1057 were taking a combination of both drugs (CAS group). Fifty-eight years old was the mean age of the study population. Notably, the average age of individuals in the CAS group (66 years) was remarkably higher than nonusers (53 years) and those using either drug alone (61 or 63 years).  Table 1 shows that individuals who utilized a combination therapy of aspirin and statins were predominantly male (58.5%), non-Hispanic White (74.5%), highly educated (58.0%), obese (45.7%), engaged in active physical activity (48.0%), former smoking (41.8%), moderate drinkers (38.5%), had HBP (75.3%), and had diabetes (39.9%) which were statistically different than other groups (*p* < 0.01).

Moreover, participants who were in the CAS group exhibited higher levels of TG (1.50 ± 0.98 mmol/L) and WC (104.7 ± 19.1 cm) than other groups but lower levels of platelets (221.1 ± 59.3∗10^9^/L) and TC (4.42 ± 0.97 mmol/L) than other groups (*p* < 0.01). Additionally, within all drug groups, the prevalence of MAFLD was 54.1%, MASLD was 54.1%, and advanced LF was 22.1%, with the highest rates observed in participants who had taken a CAS group.

### 3.2. Associations Between CAS With Prevalent MAFLD, MASLD, and Advanced LF

**Figure 2:**
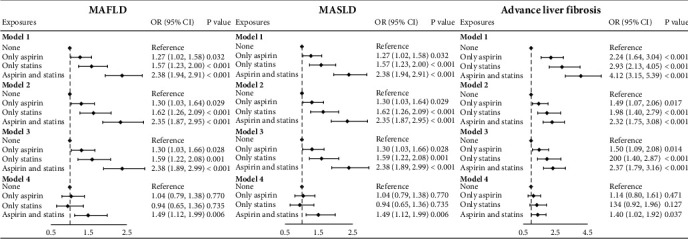
Associations between aspirin and statin use with prevalent MAFLD, MASLD, and advanced liver fibrosis; NHANES 2011–2018 (*n* = 5889). Model 1: nonadjusted model; Model 2 adjusted for gender, age, and race; Model 3 adjusted for education, poverty income ratio, smoking status, alcohol consumption, HBV infection, HCV infection, alanine aminotransferase, and physical activity status in addition to Model 2. Model 4: BMI, diabetes, and hypertriglyceridemia in addition to Model 3. Abbreviations: BMI, body mass index; HBV, hepatitis B virus; HCV, hepatitis C virus; NHANES, National Health and Nutrition Examination Survey; MAFLD, metabolic dysfunction–associated fatty liver disease; MASLD, metabolic dysfunction–associated steatotic liver disease; OR, odds ratio; 95% CI, 95% confidence interval.

The risk of MAFLD, MASLD, and advanced liver fibrosis was increased among participants with CAS, as shown in Figure 2. In Models 1, 2, and 3, single aspirin or statin use, as well as the CAS, was both positively associated with MAFLD, MASLD, or advanced LF. It was worth noting that CAS was positively associated with MAFLD, MASLD, or advanced LF, which was relatively higher than single aspirin or statin use. Notably, in the fully adjusted model (Model 4), the CAS group was the only group that was significantly associated with MAFLD, MASLD, or advanced LF (full adjustment: MAFLD and MASLD: OR = 1.49; 95% CI: 1.12–1.99; advanced LF: OR = 1.40; 95% CI: 1.02–1.92). Nevertheless, the significance of using a single drug was no longer statistically relevant when considering additional adjustments for metabolic risk factors.

### 3.3. Subgroup Analysis

**Figure 3:**
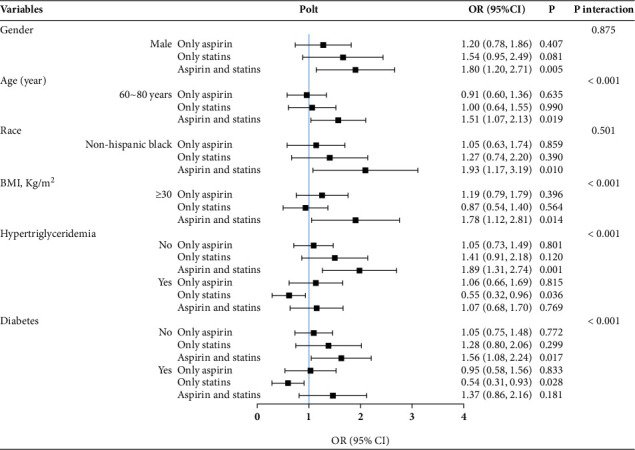
Subgroup and interaction analyses for the impact of covariates on the association of aspirin use and statin use with the prevalence of MAFLD. MAFLD, metabolic dysfunction–associated fatty liver disease; BMI, body mass index; OR, odds ratio; 95% CI, 95% confidence interval.

**Figure 4:**
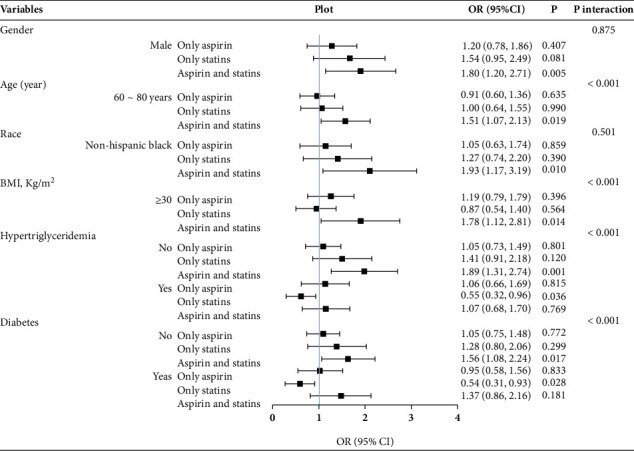
Subgroup and interaction analyses for the impact of covariates on the association of aspirin use and statin use with the prevalence of MASLD. MASLD, metabolic dysfunction–associated steatotic liver disease; BMI, body mass index; OR, odds ratio; 95% CI, 95% confidence interval.

**Figure 5:**
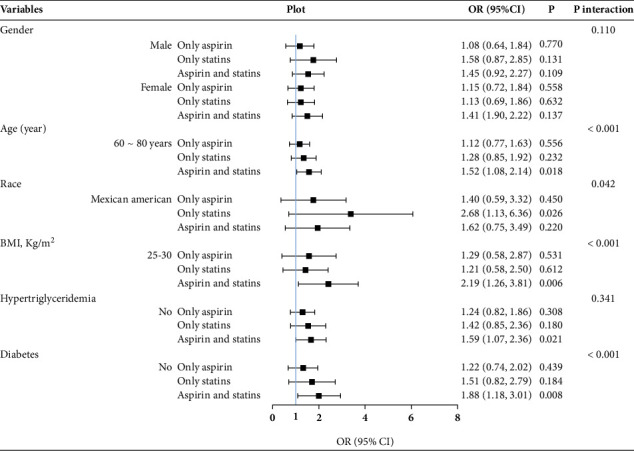
Subgroup and interaction analyses for the impact of covariates on the association of aspirin use and statin use with the prevalence of advanced liver fibrosis. BMI, body mass index; OR, odds ratio; 95% CI, 95% confidence interval.

We further studied the effect of subgroup analysis and interaction test on CAS in **Tables**S1–S3. To facilitate a comprehensive and intuitive comparison of various covariates, we presented the data in Figures 3, 4, and 5. In the full-adjusted model, the CAS was positively associated with MAFLD or MASLD among participants in men (OR = 1.80; 95% CI: 1.20–2.71), the 60–80-year-old group (OR = 1.51; 95% CI: 1.07–2.13), other Hispanics (OR = 2.97; 95% CI: 1.10–8.06), non-Hispanic Blacks (OR = 1.93; 95% CI: 1.17–3.19), BMI ≥ 30 kg/m^2^ (OR = 1.78; 95% CI: 1.12–2.81), without hypertriglyceridemia (OR = 1.89; 95% CI: 1.31–2.74), and without diabetes (OR = 1.56; 95% CI: 1.08–2.24) (Figures 3 and 4). However, among participants with hypertriglyceridemia (OR = 0.55; 95% CI: 0.32–0.96) and diabetes (OR = 0.54; 95% CI: 0.31–0.93), statin use alone was inversely associated with MAFLD or MASLD. In Figure 5, the CAS was definitely associated with advanced LF among participants in the 60–80-year-old group (OR = 1.52; 95% CI: 1.08–2.14), BMI 25–30 kg/m^2^ (OR = 2.19; 95% CI: 1.26–3.81), without hypertriglyceridemia (OR = 1.59; 95% CI: 1.07–2.36), and without diabetes (OR = 1.88; 95% CI: 1.18–3.01).

### 3.4. Interaction Analysis

Among the covariables in the full-adjusted model, it was found that age, hypertriglyceridemia, diabetes, and BMI had prominent interactions (*p* for interaction < 0.001) with CAS on MAFLD or MASLD. Furthermore, age, diabetes, and BMI had prominent interactions (*p* for interaction < 0.001) with CAS on advanced LF. However, hypertriglyceridemia was no longer the factor that interacted with CAS in relation to the risk of advanced LF.

## 4. Discussion

The study using four cycles of NHANES data found that participants of the CAS group were older, predominantly male, non-Hispanic White, and more with metabolic syndrome. Our findings indicated that aspirin or statin monotherapy did not have a significant impact on MAFLD, MASLD, or advanced LF. Surprisingly, notable positive associations between CAS and MAFLD, MASLD, or advanced LF were observed. Furthermore, different groups of age and BMI, as well as diabetes status, had significant interactions with CAS on both MAFLD/MASLD and advanced LF. These findings provided more evidence for the rational application of the CAS. For the high-risk populations, combined use should be avoided to reduce the risk of development of MASLD or advanced LF.

Based on the stratified analysis, it appears that CAS is associated with MAFLD, MASLD, or advanced LF primarily among men over 60. Older people have more metabolic disorders and physical health exams, which may account for some of this. A cross-sectional study revealed that women excreted higher levels of aspirin and its metabolites in urine 8 h after consuming 650 mg of aspirin compared to men [25]. These findings suggest that males and older individuals may have reduced metabolism and clearance of aspirin, potentially resulting in increased accumulation of the drug in the body and subsequently raising the risk of MAFLD and MASLD.

After additional adjustment for metabolic risk factors in Model 4, while aspirin or statin monotherapy showed no significant association with MAFLD, MASLD, or advanced LF, CAS was significantly associated with an increased risk of these conditions. Subgroup and interaction analyses further revealed that age, BMI, diabetes, and hypertriglyceridemia strongly modified these associations. These results highlight the critical role of pre-existing metabolic disorders as confounding factors in interpreting the relationship between CAS and liver disease. Baseline characteristics of the study population (Table 1) provide critical insights into potential confounding. The CAS group had a substantially higher prevalence of metabolic comorbidities compared to nonusers or single-drug users: 45.7% were obese (BMI ≥ 30 kg/m^2^), 39.9% had diabetes, and 75.3% had HBP. These conditions are well-established drivers of MAFLD/MASLD pathogenesis, as metabolic dysfunction—characterized by insulin resistance, dyslipidemia, and chronic low-grade inflammation—directly promotes hepatic steatosis and fibrosis. Notably, the high prevalence of these pre-existing metabolic disorders in CAS users suggests that the observed association between CAS and MAFLD/MASLD or advanced LF may not reflect a direct causal effect of the drugs themselves but rather the underlying metabolic dysfunction in this population. Clinically, aspirin is often prescribed for cardiovascular disease prevention and statins for hyperlipidemia conditions closely linked to metabolic syndrome. Thus, CAS users are inherently a high-risk group with pre-existing metabolic disturbances, which independently increase their susceptibility to liver disease.

Subgroup analyses further support the role of baseline metabolic status as a key modifier. The association between CAS and MAFLD/MASLD or advanced LF was most pronounced in populations with less severe metabolic impairment, especially in participants without diabetes or hypertriglyceridemia. In these groups, CAS was significantly associated with higher risks of MAFLD/MASLD and advanced LF. This may be because individuals with milder metabolic dysfunction have less severe underlying liver pathology, making the additive effect of CAS (and its potential interplay with subclinical metabolic disturbances) more detectable. CAS was associated with a 78% higher risk of MAFLD/MASLD in overweight/obese individuals (BMI ≥ 25 kg/m^2^). While obesity itself is a strong risk factor for liver disease, the CAS may exacerbate the metabolic burden in these individuals, possibly through unmeasured factors such as cumulative drug exposure or interactions with adiposity-related inflammation. In contrast, among participants with diabetes or hypertriglyceridemia, statin monotherapy was inversely associated with MAFLD/MASLD. This may reflect the fact that in populations with severe metabolic dysfunction, the primary driver of liver disease is the underlying metabolic disorder itself, overshadowing any potential effect of drug use. Additionally, statins may exert modest protective effects in these groups by improving lipid profiles, a benefit that could counteract their potential hepatotoxicity in the context of severe dyslipidemia. This indicates that statins show promise in the treatment of MAFLD/MASLD, which is consistent with previous studies [26].

The previously unreported finding that overweight/obese individuals without diabetes or hypertriglyceridemia showed a significant association with CAS and MAFLD, MASLD, or advanced LF may be attributed to the unclear toxicity of drug use in combination also. While the exact pathogenesis of MAFLD and MASLD remains uncertain, the “multiple hit theory” has been widely embraced. This theory posits that oxidative stress resulting from mitochondrial dysfunction plays a vital role in exacerbating liver cell apoptosis and necrosis, stimulating hepatic stellate cell activation for collagen deposition, and eventually leading to liver fibrosis [27]. As a general rule, aspirin demonstrates cytotoxic and anti-inflammatory properties through a variety of mechanisms, such as mitochondrial dysfunction, reactive oxygen species production, and apoptosis induction [28–30]. Furthermore, there is ongoing debate concerning the efficacy of statins in treating MAFLD patients due to concerns about potential liver dysfunction and muscle-related side effects. The precise mechanism by which statins induce liver damage remains unclear, but it is hypothesized that mitochondrial dysfunction resulting in cellular apoptosis may play a role. A recent study has shown that low doses of statins can elevate intracellular calcium levels, leading to mitochondrial dysfunction and apoptosis in HepG2 cells [31].

The primary goal of treating liver disease in patients with MAFLD or MASLD is to arrest the advancement of hepatic fibrosis and reduce the likelihood of developing cirrhosis and cancer formation. The leading cause of mortality in MASLD patients is systemic metabolic dysfunction, notably cardiovascular disease [32]. Among pharmacological interventions that target cardiovascular disease risk factors effectively, aspirin and statins play an important role. This study identified a significant association between CAS and an increased risk of MAFLD, MASLD, or advanced LF. Collectively, these findings indicate that the use of CAS requires vigilant monitoring for hepatotoxicity and adverse effects, and further investigation is warranted to clarify the underlying mechanisms.

The findings underscore the importance of considering baseline metabolic status when prescribing CAS. Clinicians should exercise caution when combining aspirin and statins in individuals with mild to moderate metabolic dysfunction (e.g., overweight/obese without diabetes or hypertriglyceridemia), as these individuals may be more vulnerable to the additive risks of CAS on liver health. In contrast, in patients with established diabetes or hypertriglyceridemia, the benefits of statins (e.g., lipid control) may outweigh potential risks, and the primary focus should remain on managing the underlying metabolic disorder. Notably, the lack of association between single-drug use and liver disease after adjusting for metabolic factors suggests that aspirin or statins alone are not independently harmful in the context of controlled metabolic status. This aligns with clinical guidelines supporting their use for cardiovascular and lipid management, provided that metabolic comorbidities are well-controlled.

This study was limited in all of the following aspects. First, as a retrospective cross-sectional analysis based on existing NHANES data, this study is inherently subject to selection bias and residual confounding. For example, unmeasured factors such as duration of aspirin/statin use, dosage adjustments, and adherence to medication (which may differ between CAS users and nonusers) could not be fully controlled, potentially affecting the observed associations. Additionally, the retrospective design prevents us from inferring causality, as we cannot exclude the possibility that pre-existing metabolic dysfunction in CAS users (e.g., more severe insulin resistance or subclinical liver steatosis) predated drug use and contributed to the observed risk. In the future, prospective cohort studies should be conducted to clarify the causal relationship between CAS and MASLD and to systematically adjust for all potential confounding factors, or to clarify the temporal relationship between CAS, metabolic status, and liver disease progression.

## 5. Conclusion

**Figure 6:**
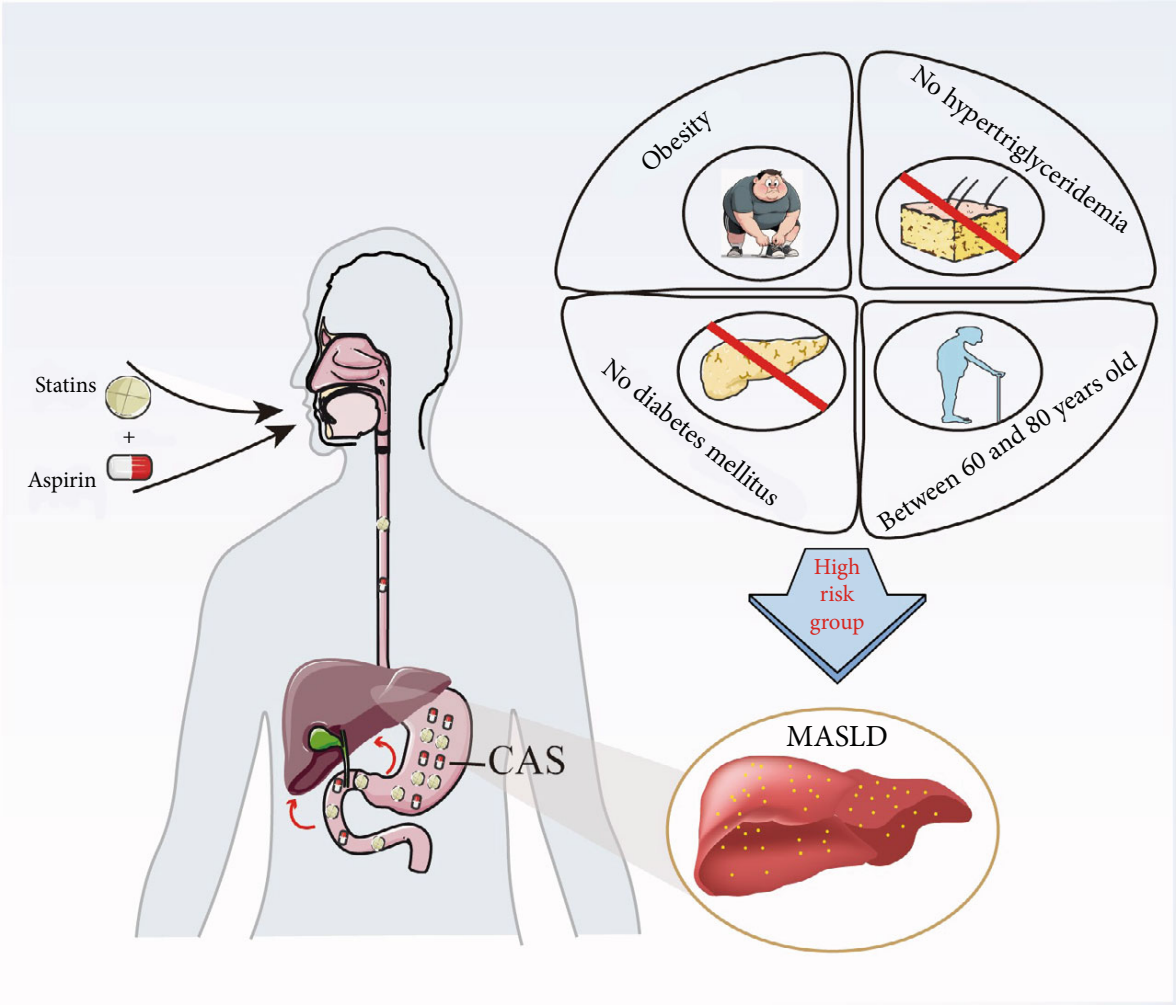
Combined use of aspirin and statins was associated with the risk of MASLD, especially for those who are people over 60 years old, those who are overweight, or those without diabetes/hypertriglyceridemia. CAS, combined use of aspirin and statins; MASLD, metabolic dysfunction–associated steatotic liver disease.

In summary, the current study's findings indicate that CAS may elevate the likelihood of developing MAFLD, MASLD, or advanced LF. Consequently, individuals with MASLD should exercise caution when contemplating the utilization of CAS, especially for persons between 60 and 80 years old, of overweight, without diabetes, or without hypertriglyceridemia (Figure 6). Moreover, additional prospective cohort investigations and randomized controlled trials are necessary to investigate and validate these findings.

## Figures and Tables

**Table 1:** General characteristics of included participants (*n* = 5889) by the presence or absence of aspirin and statin use in the NHANES 2011–2018.

	**Total**	**None**	**Only aspirin**	**Only statins**	**Aspirin and statins**	**p** ** value**
Sample *N* (%)	5889	3222 (55.4)	903 (15.6)	707 (11.9)	1057 (17.0)	—
Age (years)	57.9 ± 11.4	53.4 ± 10.3	61.3 ± 10.3	62.6 ± 10.2	65.7 ± 9.7	< 0.001
40~59	57.5 (55.7–59.3)	74.3 (72.1–76.3)	43.2 (38.6–48.0)	41.5 (36.2–47.0)	27.5 (23.6–31.8)	
60~80	42.5 (40.7–44.3)	25.7 (23.7–27.9)	56.8 (52.0–61.4)	58.5 (53.0–63.8)	72.5 (68.2–76.4)	
Gender						< 0.001
Male	47.9 (46.0–49.7)	45.9 (43.4–48.4)	45.8 (41.2–50.5)	44.6 (39.3–50.0)	58.5 (54.2–62.7)	
Female	52.1 (50.3–54)	54.1 (51.6–56.6)	54.2 (49.5–58.8)	55.4 (50.0–60.7)	41.5 (37.3–45.8)	
Race/ethnicity						< 0.001
Mexican American	6.6 (6.1–7.2)	8.4 (7.6–9.3)	4.4 (3.5–5.7)	4.8 (3.7–6.2)	3.9 (3.1–5.0)	
Other Hispanic	5.6 (5.1–6.1)	6.6 (5.9–7.4)	4.9 (3.8–6.2)	4.1 (3.2–5.4)	3.8 (3.0–4.7)	
Non-Hispanic White	69.4 (68.1–70.8)	65.4 (63.4–67.4)	75.1 (72.1–77.9)	73.6 (69.6–77.2)	74.5 (71.5–77.4)	
Non-Hispanic Black	10.2 (9.6–10.9)	10.7 (9.8–11.7)	10.5 (9.0–12.4)	8.9 (7.3–10.8)	9.5 (8.2–11.1)	
Other races^a^	8.1 (7.4–8.9)	8.9 (7.9–10.0)	5.0 (4.0–6.3)	8.6 (6.3–11.6)	8.2 (6.4–10.6)	
Education						< 0.001
More than high school	61.4 (59.6–63.1)	63.2 (60.9–65.5)	62.8 (58.2–67.1)	55.8 (50.3–61.1)	58.0 (53.8–62.2)	
High school or equivalent	22.7 (21.2–24.3)	20.2 (18.3–22.3)	24.9 (21.0–29.4)	26.3 (21.6–31.7)	26.4 (22.8–30.5)	
Less than high school	15.9 (14.8–17.0)	16.6 (15.1–18.2)	12.2 (10.1–14.7)	17.9 (14.4–21.9)	15.5 (13.2–18.2)	
Not recorded	0.0 (0.0–0.1)	0.0 (0.0–0.1)	0.1 (0.0–0.4)	0.0 (0.0–0.0)	0.0 (0.0–0.1)	
Poverty–income ratio						0.355
< 1.3	16.9 (15.9–18.1)	17.7 (16.2–19.2)	16.2 (13.6–19.2)	18.4 (14.8–22.7)	14.3 (12.1–16.8)	
1.3–1.8	8.6 (7.7–9.5)	8.5 (7.4–9.8)	9.0 (7.1–11.5)	7.7 (5.6–10.4)	8.8 (7.1–11.0)	
> 1.8	66.7 (65.1–68.3)	66.3 (64.1–68.4)	66.9 (62.8–70.9)	66.7 (61.7–71.3)	68.0 (64.2–71.5)	
Not recorded	7.8 (6.9–8.8)	7.5 (6.4–8.8)	7.8 (5.7–10.6)	7.2 (5.2–10.0)	8.9 (6.7–11.8)	
BMI group (kg/m^2^)						< 0.001
< 20	3.2 (2.5–3.9)	3.4 (2.6–4.6)	4.0 (2.3–6.9)	1.9 (0.9–4.0)	2.4 (1.3–4.3)	
20–25	21.5 (20.0–23.1)	23.6 (21.6–25.8)	18.1 (14.9–21.7)	20.3 (16.1–25.4)	18.5 (15.4–22.1)	
25–30	34.6 (32.9–36.4)	35.2 (32.9–37.6)	36.7 (32.2–41.4)	31.8 (27.2–36.9)	32.9 (29.0–36.9)	
≥ 30	40.3 (38.5–42.1)	37.4 (35.0–39.8)	40.6 (36.1–45.3)	45.8 (40.4–51.3)	45.7 (41.3–50.1)	
Not recorded	0.4 (0.3–0.6)	0.3 (0.2–0.6)	0.6 (0.3–1.4)	0.2 (0.0–0.8)	0.6 (0.3–1.1)	
Physical activity level						< 0.001
Active	53.6 (51.7–55.4)	56.9 (54.5–59.3)	52.4 (47.7–57.1)	47.6 (42.2–53.1)	48.0 (43.7–52.4)	
Less active	15.5 (14.2–17.0)	15.4 (13.7–17.3)	15.0 (11.7–19.1)	17.8 (14.0–22.4)	14.8 (11.9–18.1)	
Inactive	30.8 (29.2–32.4)	27.6 (25.6–29.7)	32.5 (28.3–36.9)	34.5 (29.8–39.6)	37.1 (33.1–41.2)	
Not recorded	0.1 (0.1–0.2)	0.1 (0.0–0.3)	0.1 (0.0–0.4)	0.1 (0.0–0.4)	0.1 (0.0–0.6)	
Smoking status						< 0.001
Never	52.4 (50.6–54.3)	56.2 (53.8–58.7)	51.1 (46.4–55.8)	48.8 (43.4–54.2)	43.8 (39.6–48.2)	
Former	30.6 (28.9–32.3)	25.7 (23.6–28.0)	32.7 (28.4–37.2)	34.2 (29.4–39.4)	41.8 (37.5–46.2)	
Current	17.0 (15.6–18.4)	18.0 (16.2–19.9)	16.2 (13.1–19.9)	17.0 (13.1–21.9)	14.3 (11.6–17.7)	
Not recorded	0.0 (0.0–0.1)	0.1 (0.0–0.2)	0.0 (0.0–0.0)	0.0 (0.0–0.0)	0.0 (0.0–0.1)	
Alcohol consumption						< 0.001
Never	9.7 (8.8–10.7)	9.8 (8.7–11.1)	10.9 (8.3–14.2)	9.3 (7.1–12.2)	8.7 (6.8–10.9)	
Moderate	33.9 (32.1–35.7)	32.4 (30.0–34.8)	33.7 (29.4–38.3)	34.7 (29.6–40.3)	38.5 (34.3–42.9)	
Heavy	38.4 (36.6–40.3)	41.7 (39.2–44.2)	37.0 (32.5–41.8)	35.0 (29.9–40.4)	31.6 (27.6–36.0)	
Not recorded	17.9 (16.7–19.2)	16.1 (14.6–17.8)	18.4 (15.3–22.0)	21.0 (17.2–25.3)	21.2 (18.1–24.7)	
Hypertension						< 0.001
Yes	54.0 (52.2–55.9)	43.2 (40.7–45.6)	62.0 (57.2–66.6)	63.8 (58.1–69.0)	75.3 (71.2–78.9)	
History of diabetes						< 0.001
Yes	20.3 (18.9–21.7)	10.7 (9.5–12.1)	23.1 (19.6–27.0)	32.7 (28.0–37.7)	39.9 (35.8–44.1)	
Having HBV infection						0.028
Yes	0.8 (0.5–1.2)	0.7 (0.5–1.1)	0.3 (0.1–0.6)	0.6 (0.2–1.6)	1.6 (0.5–4.8)	
Having HCV infection						0.003
Yes	2.2 (1.6–2.9)	2.7 (1.9–3.8)	2.5 (1.3–4.6)	1.3 (0.3–5.1)	0.8 (0.3–1.8)	
Laboratory parameters						
ALT (U/L)	24.6 ± 15.6	24.9 ± 17.0	24.2 ± 16.3	24.1 ± 11.9	24.1 ± 12.5	0.385
AST (U/L)	25.0 ± 16.1	25.1 ± 18.4	25.1 ± 17.1	24.3 ± 8.9	24.7 ± 9.3	0.574
GGT (IU/L)	29.9 ± 35.5	30.2 ± 38.3	27.8 ± 27.6	31.7 ± 37.1	29.5 ± 31.0	0.137
Albumin (g/L)	41.9 ± 3.2	41.9 ± 3.3	41.7 ± 3.1	41.6 ± 3.2	41.9 ± 3.2	0.042
Total cholesterol (mmol/L)	5.07 ± 1.08	5.28 ± 0.98	5.38 ± 1.10	4.65 ± 1.13	4.42 ± 0.97	< 0.001
Triglyceride (mmol/L)	1.42 ± 1.14	1.36 ± 0.96	1.48 ± 1.06	1.50 ± 1.93	1.50 ± 0.98	< 0.001
Platelet (∗10^9^/L)	231.3 ± 59.5	234.7 ± 58.9	233.4 ± 62.0	226.9 ± 57.1	221.1 ± 59.3	< 0.001
Waist circumference (cm)	101.3 ± 18.7	99.5 ± 18.0	102.1 ± 19.9	103.9 ± 18.3	104.7 ± 19.1	< 0.001
MAFLD						< 0.001
Yes	38.9 (37.1–40.7)	33.2 (30.9–35.6)	38.7 (34.3–43.4)	43.8 (38.5–49.2)	54.1 (49.8–58.4)	
MASLD						< 0.001
Yes	38.9 (37.1–40.7)	33.2 (30.9–35.6)	38.7 (34.3–43.4)	43.8 (38.5–49.2)	54.1 (49.8–58.4)	
Advanced liver fibrosis						< 0.001
Yes	11.4 (10.4–12.5)	6.4 (5.4–7.7)	13.3 (10.7–16.4)	16.8 (13.5–20.7)	22.1 (19.0–25.5)	

*Note:* Values are weighted mean ± SE or weighted% (95% confidence interval). *p* values are weighted.

Abbreviations: ALT, alanine aminotransferase; AST, aspartate aminotransferase; BMI, body mass index; GGT, gamma glutamyl transferase; HDL, high density lipoprotein; MAFLD, metabolic dysfunction–associated fatty liver disease; MASLD, metabolic dysfunction–associated steatotic liver disease; NHANES, National Health and Nutrition Examination Survey.

^a^Other races include Asian, American Indian or Alaska Native, Native Hawaiian or other Pacific Islander, and multiracial persons.

## Data Availability

Publicly available datasets were analyzed in this study. This data can be found here: https://wwwn.cdc.gov/nchs/nhanes/.

## Nomenclature

ALB: albumin
ALT: alanine aminotransferase
AST: aspartate aminotransferase
APRI: aminotransferase-to-platelet ratio index
BMI: body mass index
CAS: combined use of aspirin and statins
FIB-4: fibrosis index based on four factors
GGT: gamma glutamyl transferase
HbA1c: glycohemoglobin
HBV: hepatitis B virus
HBP: hypertension
HCV: hepatitis C virus
HDL-C: high-density lipoprotein cholesterol
MAFLD: metabolic dysfunction–associated fatty liver disease
MASLD: metabolic dysfunction-associated steatotic liver disease
NASH: nonalcoholic steatohepatitis
NFS: nonalcoholic fatty liver disease fibrosis score
NHANES: National Health and Nutrition Examination Survey
PLT: platelet
T2DM: Type 2 diabetic mellitus
TC: total cholesterol
TGs: triglycerides
US-FLI: US-Fatty Liver Index

## Supporting Information

Supporting Information Additional supporting information can be found online in the Supporting Information section. Subgroup analysis and interaction test results on CAS with liver diseases could be found in Tables S1–S3.
