# Supplementary material for: Combined Use of Aspirin and Statins Is Associated With Increased Risk of Metabolic Dysfunction–Associated Steatotic Liver Disease: A Study From National Health and Nutrition Examination Survey
Source: Gastroenterol Res Pract. 2025 Oct 5;2025:9931208. doi: 10.1155/grp/9931208 (PMC12515564; doi:10.1155/grp/9931208)
Supplement: Supporting Information — Additional supporting information can be found online in the Supporting Information section. Subgroup analysis and interaction test results on CAS with liver diseases could be found in Tables S1–S3. [file 9931208.f1.docx]

**Supplementary table 1 |** Subgroup and interaction analyses for the impact of covariates on the association of aspirin use and statins use with the prevalent of MAFLD.

|  |  |  | **Model1 OR (95%CI), *P*** | **Model2 OR (95%CI), *P*** | **Model3 OR (95%CI), *P*** | **Model4 OR (95%CI), *P*** |
| --- | --- | --- | --- | --- | --- | --- |
| **MAFLD** |  |  |  |  |  |  |
| **Gender** | **Male** | Aspirin | 1.25(0.91,1.71) 0.168 | 1.29(0.92,1.80) 0.136 | 1.24(0.88,1.74) 0.216 | 1.20(0.78,1.86) 0.407 |
|  |  | Statins | 1.75(1.23,2.49) 0.002 | 1.79(1.23,2.60) 0.002 | 1.87(1.27,2.74) 0.001 | 1.54(0.95,2.49) 0.081 |
|  |  | Aspirin and statins | 2.49(1.87,3.30)＜0.001 | 2.60(1.89,3.57)＜0.001 | 2.72(1.97,3.77)＜0.001 | 1.80(1.20,2.71)0.005 |
|  | **Female** | Aspirin | 1.30(0.96,1.76) 0.092 | 1.31(0.94,1.82) 0.107 | 1.34(0.96,1.87) 0.087 | 0.92(0.64,1.32) 0.650 |
|  |  | Statins | 1.45(1.04,2.02) 0.027 | 1.48(1.04,2.10) 0.028 | 1.39(0.96,1.99) 0.079 | 0.67(0.40,1.14) 0.138 |
|  |  | Aspirin and statins | 2.02(1.51,2.71)＜0.001 | 2.06(1.49,2.85)＜0.001 | 1.99(1.44,2.77)＜0.001 | 1.22(0.80,1.85) 0.356 |
| **Age (years)** | **40~59** | Aspirin | 1.57(1.13,2.17) 0.007 | 1.64(1.16,2.30) 0.005 | 1.66(1.16,2.36) 0.005 | 1.28(0.88,1.85) 0.199 |
|  |  | Statins | 1.69(1.16,2.47) 0.006 | 1.78(1.21,2.60) 0.003 | 1.75(1.16,2.63) 0.008 | 0.84(0.45,1.56) 0.578 |
|  |  | Aspirin and statins | 2.06(1.41,3.01)＜0.001 | 2.09(1.41,3.09)＜0.001 | 2.13(1.42,3.19)＜0.001 | 1.35(0.80,2.31) 0.264 |
|  | **60~80** | Aspirin | 1.02(0.74,1.39) 0.916 | 1.03(0.74,1.42) 0.879 | 1.08(0.78,1.50) 0.653 | 0.91(0.60,1.36) 0.635 |
|  |  | Statins | 1.40(1.00,1.96) 0.052 | 1.43(1.01,2.03) 0.041 | 1.44(1.01,2.04) 0.043 | 1.00(0.64,1.55) 0.990 |
|  |  | Aspirin and statins | 2.34(1.77,3.08)＜0.001 | 2.30(1.73,3.06)＜0.001 | 2.38(1.78,3.19)＜0.001 | 1.51(1.07,2.13) 0.019 |
| **Race** | **Mexican American** | Aspirin | 1.50(0.88,2.57) 0.139 | 1.49(0.87,2.56) 0.151 | 1.75(0.99,3.09) 0.054 | 1.67(0.89,3.13) 0.111 |
|  |  | Statins | 2.00(1.11,3.59) 0.021 | 1.97(1.09,3.59) 0.026 | 2.23(1.21,4.11) 0.010 | 1.25(0.58,2.71) 0.565 |
|  |  | Aspirin and statins | 2.14(1.19,3.83) 0.011 | 2.04(1.08,3.86) 0.028 | 2.61(1.38,4.94) 0.003 | 1.50(0.73,3.10) 0.268 |
|  | **Other Hispanic** | Aspirin | 2.27(1.35,3.83) 0.002 | 2.36(1.37,4.07) 0.002 | 2.79(1.61,4.84)＜0.001 | 2.67(1.39,5.14) 0.003 |
|  |  | Statins | 2.20(1.24,3.91) 0.007 | 2.41(1.34,4.33) 0.003 | 2.40(1.25,4.62) 0.009 | 1.00(0.39,2.59) 0.992 |
|  |  | Aspirin and statins | 3.27(2.00,5.33)＜0.001 | 3.52(2.11,5.87)＜0.001 | 4.26(2.40,7.54)＜0.001 | 2.97(1.10,8.06) 0.032 |
|  | **Non-Hispanic White** | Aspirin | 1.23(0.92,1.64) 0.166 | 1.19(0.88,1.61) 0.269 | 1.19(0.87,1.62) 0.278 | 0.96(0.66,1.39) 0.828 |
|  |  | Statins | 1.52(1.11,2.10) 0.010 | 1.47(1.06,2.04) 0.022 | 1.44(1.02,2.03) 0.037 | 0.87(0.54,1.41) 0.580 |
|  |  | Aspirin and statins | 2.40(1.84,3.13)＜0.001 | 2.13(1.59,2.84)＜0.001 | 2.10(1.56,2.82)＜0.001 | 1.33(0.92,1.93) 0.131 |
|  | **Non-Hispanic Black** | Aspirin | 1.36(0.91,2.03) 0.131 | 1.46(0.97,2.19) 0.070 | 1.42(0.92,2.18) 0.111 | 1.05(0.63,1.74) 0.859 |
|  |  | Statins | 2.19(1.43,3.36)＜0.001 | 2.39(1.54,3.70)＜0.001 | 2.55(1.60,4.05)＜0.001 | 1.27(0.74,2.20) 0.390 |
|  |  | Aspirin and statins | 2.61(1.81,3.75)＜0.001 | 2.98(2.01,4.43)＜0.001 | 3.23(2.15,4.84)＜0.001 | 1.93(1.17,3.19) 0.010 |
|  | **Other Races1** | Aspirin | 1.68(1.00,2.84) 0.050 | 1.62(0.94,2.82) 0.085 | 1.53(0.84,2.78) 0.161 | 1.17(0.61,2.26) 0.640 |
|  |  | Statins | 1.74(0.95,3.18) 0.074 | 1.74(0.94,3.24) 0.080 | 1.59(0.79,3.17) 0.192 | 1.12(0.39,3.19) 0.832 |
|  |  | Aspirin and statins | 3.58(2.01,6.37)＜0.001 | 3.39(1.83,6.28)＜0.001 | 3.41(1.87,6.22)＜0.001 | 2.23(0.89,5.60) 0.087 |
| **BMI group (kg/m**2)** | **20–25** | Aspirin | 1.46(0.63,3.34) 0.376 | 1.66(0.70,3.93) 0.251 | 1.52(0.68,3.38) 0.306 | 1.41(0.62,3.20) 0.409 |
|  |  | Statins | 3.10(1.33,7.22) 0.009 | 3.45(1.44,8.27) 0.005 | 3.26(1.39,7.66) 0.007 | 2.46(0.96,6.32) 0.062 |
|  |  | aspirin and statins | 2.56(1.27,5.18) 0.009 | 2.73(1.32,5.62) 0.006 | 2.72(1.24,5.99) 0.013 | 1.42(0.53,3.86) 0.488 |
|  | **25–30** | Aspirin | 1.03(0.69,1.53) 0.888 | 0.97(0.64,1.48) 0.898 | 1.06(0.70,1.62) 0.775 | 0.91(0.58,1.42) 0.667 |
|  |  | Statins | 1.31(0.86,1.99 )0.215 | 1.24(0.79,1.96) 0.355 | 1.30(0.82,2.08) 0.269 | 0.78(0.45,1.35) 0.374 |
|  |  | Aspirin and statins | 2.15(1.51,3.07)＜0.001 | 1.92(1.28,2.89) 0.002 | 2.04(1.36,3.06) 0.001 | 1.27(0.83,1.95) 0.266 |
|  | **≥30** | Aspirin | 1.35(0.95,1.93) 0.093 | 1.43(0.97,2.09) 0.071 | 1.46(0.98,2.17) 0.062 | 1.19(0.79,1.79) 0.396 |
|  |  | Statins | 1.32(0.89,1.95) 0.168 | 1.33(0.88,2.02) 0.173 | 1.28(0.82,2.02) 0.279 | 0.87(0.54,1.40) 0.564 |
|  |  | Aspirin and statins | 3.23(2.22,4.71)＜0.001 | 2.75(1.79,4.24)＜0.001 | 2.83(1.84,4.36)＜0.001 | 1.78(1.12,2.81) 0.014 |
| **Hypertriglyceridemia** | **no** | Aspirin | 1.15(0.87,1.52) 0.336 | 1.20(0.89,1.63) 0.230 | 1.26(0.92,1.71) 0.150 | 1.05(0.73,1.49) 0.801 |
|  |  | Statins | 1.77(1.28,2.44) 0.001 | 1.90(1.36,2.67)＜0.001 | 1.96(1.40,2.76)＜0.001 | 1.41(0.91,2.18) 0.120 |
|  |  | Aspirin and statins | 2.54(1.94,3.33)＜0.001 | 2.58(1.91,3.50)＜0.001 | 2.57(1.89,3.50)＜0.001 | 1.89(1.31,2.74) 0.001 |
|  | **yes** | Aspirin | 1.32(0.87,2.00) 0.199 | 1.20(0.89,1.63) 0.230 | 1.22(0.79,1.87) 0.368 | 1.06(0.66,1.69) 0.815 |
|  |  | Statins | 0.80(0.54,1.19)0.266 | 1.90(1.36,2.67)＜0.001 | 0.73(0.47,1.13)0.155 | 0.55(0.32,0.96)0.036 |
|  |  | Aspirin and statins | 1.22(0.87,1.71)0.254 | 2.58(1.91,3.50)＜0.001 | 1.14(0.78,1.67)0.486 | 1.07(0.68,1.70)0.769 |
| **Diabetes** | **no** | Aspirin | 0.98(0.74,1.29)0.874 | 0.99(0.73,1.34)0.944 | 1.04(0.77,1.40)0.815 | 1.05(0.75,1.48)0.772 |
|  |  | Statins | 1.42(1.03,1.96)0.032 | 1.49(1.07,2.08)0.019 | 1.47(1.04,2.06)0.028 | 1.28(0.80,2.06)0.299 |
|  |  | Aspirin and statins | 1.74(1.31,2.32)＜0.001 | 1.69(1.23,2.31)0.001 | 1.73(1.26,2.39)0.001 | 1.56(1.08,2.24)0.017 |
|  | yes | Aspirin | 1.09(0.70,1.68)0.712 | 1.16(0.73,1.83)0.535 | 1.04(0.65,1.67)0.869 | 0.95(0.58,1.56)0.833 |
|  |  | Statins | 0.68(0.44,1.05)0.082 | 0.67(0.42,1.07)0.092 | 0.63(0.40,1.02)0.059 | 0.54(0.31,0.93)0.028 |
|  |  | Aspirin and statins | 1.24(0.85,1.81)0.269 | 1.42(0.93,2.17)0.108 | 1.43(0.94,2.18)0.093 | 1.37(0.86,2.16)0.181 |

**Note:** Model 1: Non-adjusted model; Model 2 adjusted for: gender; age; race; Model 3 adjusted for: education, poverty income ratio, smoking status, alcohol consumption; HBV infection; HCV infection; alanine aminotransferase, and physical activity status in addition to model 2. Model 4: BMI, diabetes, hypertriglyceridemia in addition to model 3.

**Abbreviations:** MAFLD, metabolic dysfunction-associated fatty liver disease; OR, odds ratio; 95%CI, 95% confidence interval.

**Supplementary table 2 |** Subgroup and interaction analyses for the impact of covariates on the association of aspirin use and statins use with the prevalent of MASLD.

|  |  |  | **Model1 OR (95%CI), *P*** | **Model2 OR (95%CI), *P*** | **Model3 OR (95%CI), *P*** | **Model4 OR (95%CI), *P*** |
| --- | --- | --- | --- | --- | --- | --- |
| **MASLD** |  |  |  |  |  |  |
| **Gender** | **Male** | Aspirin | 1.25(0.91,1.71) 0.168 | 1.29(0.92,1.80) 0.136 | 1.24(0.88,1.74) 0.216 | 1.20(0.78,1.86) 0.407 |
|  |  | Statins | 1.75(1.23,2.49) 0.002 | 1.79(1.23,2.60) 0.002 | 1.87(1.27,2.74) 0.001 | 1.54(0.95,2.49) 0.081 |
|  |  | Aspirin and statins | 2.49(1.87,3.30)＜0.001 | 2.60(1.89,3.57)＜0.001 | 2.72(1.97,3.77)＜0.001 | 1.80(1.20,2.71) 0.005 |
|  | **Female** | Aspirin | 1.30(0.96,1.76) 0.092 | 1.31(0.94,1.82) 0.107 | 1.34(0.96,1.87) 0.087 | 0.92(0.64,1.32) 0.650 |
|  |  | Statins | 1.45(1.04,2.02) 0.027 | 1.48(1.04,2.10) 0.028 | 1.39(0.96,1.99) 0.079 | 0.67(0.40,1.14) 0.138 |
|  |  | Aspirin and statins | 2.02(1.51,2.71)＜0.001 | 2.06(1.49,2.85)＜0.001 | 1.99(1.44,2.77)＜0.001 | 1.22(0.80,1.85) 0.356 |
| **Age (years)** | **40~59** | Aspirin | 1.57(1.13,2.17) 0.007 | 1.64(1.16,2.30) 0.005 | 1.66(1.16,2.36) 0.005 | 1.28(0.88,1.85) 0.199 |
|  |  | Statins | 1.69(1.16,2.47) 0.006 | 1.78(1.21,2.60) 0.003 | 1.75(1.16,2.63) 0.008 | 0.84(0.45,1.56) 0.578 |
|  |  | Aspirin and statins | 2.06(1.41,3.01)＜0.001 | 2.09(1.41,3.09)＜0.001 | 2.13(1.42,3.19)＜0.001 | 1.35(0.80,2.31) 0.264 |
|  | **60~80** | Aspirin | 1.02(0.74,1.39) 0.916 | 1.03(0.74,1.42) 0.879 | 1.08(0.78,1.50) 0.653 | 0.91(0.60,1.36) 0.635 |
|  |  | Statins | 1.40(1.00,1.96) 0.052 | 1.43(1.01,2.03) 0.041 | 1.44(1.01,2.04) 0.043 | 1.00(0.64,1.55) 0.990 |
|  |  | Aspirin and statins | 2.34(1.77,3.08)＜0.001 | 2.30(1.73,3.06)＜0.001 | 2.38(1.78,3.19)＜0.001 | 1.51(1.07,2.13) 0.019 |
| **Race** | **Mexican American** | Aspirin | 1.50(0.88,2.57) 0.139 | 1.49(0.87,2.56) 0.151 | 1.75(0.99,3.09) 0.054 | 1.67(0.89,3.13) 0.111 |
|  |  | Statins | 2.00(1.11,3.59) 0.021 | 1.97(1.09,3.59) 0.026 | 2.23(1.21,4.11) 0.010 | 1.25(0.58,2.71) 0.565 |
|  |  | Aspirin and statins | 2.14(1.19,3.83) 0.011 | 2.04(1.08,3.86) 0.028 | 2.61(1.38,4.94) 0.003 | 1.50(0.73,3.10) 0.268 |
|  | **Other Hispanic** | Aspirin | 2.27(1.35,3.83) 0.002 | 2.36(1.37,4.07) 0.002 | 2.79(1.61,4.84)＜0.001 | 2.67(1.39,5.14) 0.003 |
|  |  | Statins | 2.20(1.24,3.91) 0.007 | 2.41(1.34,4.33) 0.003 | 2.40(1.25,4.62) 0.009 | 1.00(0.39,2.59) 0.992 |
|  |  | Aspirin and statins | 3.27(2.00,5.33)＜0.001 | 3.52(2.11,5.87)＜0.001 | 4.26(2.40,7.54)＜0.001 | 2.97(1.10,8.06) 0.032 |
|  | **Non-Hispanic White** | Aspirin | 1.23(0.92,1.64) 0.166 | 1.19(0.88,1.61) 0.269 | 1.19(0.87,1.62) 0.278 | 0.96(0.66,1.39) 0.828 |
|  |  | Statins | 1.52(1.11,2.10) 0.010 | 1.47(1.06,2.04) 0.022 | 1.44(1.02,2.03) 0.037 | 0.87(0.54,1.41) 0.580 |
|  |  | Aspirin and statins | 2.40(1.84,3.13)＜0.001 | 2.13(1.59,2.84)＜0.001 | 2.10(1.56,2.82)＜0.001 | 1.33(0.92,1.93) 0.131 |
|  | **Non-Hispanic Black** | Aspirin | 1.36(0.91,2.03) 0.131 | 1.46(0.97,2.19) 0.070 | 1.42(0.92,2.18) 0.111 | 1.05(0.63,1.74) 0.859 |
|  |  | Statins | 2.19(1.43,3.36)＜0.001 | 2.39(1.54,3.70)＜0.001 | 2.55(1.60,4.05)＜0.001 | 1.27(0.74,2.20) 0.390 |
|  |  | Aspirin and statins | 2.61(1.81,3.75)＜0.001 | 2.98(2.01,4.43)＜0.001 | 3.23(2.15,4.84)＜0.001 | 1.93(1.17,3.19) 0.010 |
|  | **Other Races1** | Aspirin | 1.68(1.00,2.84) 0.050 | 1.62(0.94,2.82) 0.085 | 1.53(0.84,2.78) 0.161 | 1.17(0.61,2.26) 0.640 |
|  |  | Statins | 1.74(0.95,3.18) 0.074 | 1.74(0.94,3.24) 0.080 | 1.59(0.79,3.17) 0.192 | 1.12(0.39,3.19) 0.832 |
|  |  | Aspirin and statins | 3.58(2.01,6.37)＜0.001 | 3.39(1.83,6.28)＜0.001 | 3.41(1.87,6.22)＜0.001 | 2.23(0.89,5.60) 0.087 |
| **BMI group (kg/m**2)** | **20–25** | Aspirin | 1.46(0.63,3.34) 0.376 | 1.66(0.70,3.93) 0.251 | 1.52(0.68,3.38) 0.306 | 1.41(0.62,3.20) 0.409 |
|  |  | Statins | 3.10(1.33,7.22) 0.009 | 3.45(1.44,8.27) 0.005 | 3.26(1.39,7.66) 0.007 | 2.46(0.96,6.32) 0.062 |
|  |  | aspirin and statins | 2.56(1.27,5.18) 0.009 | 2.73(1.32,5.62) 0.006 | 2.72(1.24,5.99) 0.013 | 1.42(0.53,3.86) 0.488 |
|  | **25–30** | Aspirin | 1.03(0.69,1.53) 0.888 | 0.97(0.64,1.48) 0.898 | 1.06(0.70,1.62) 0.775 | 0.91(0.58,1.42) 0.667 |
|  |  | Statins | 1.31(0.86,1.99) 0.215 | 1.24(0.79,1.96) 0.355 | 1.30(0.82,2.08) 0.269 | 0.78(0.45,1.35) 0.374 |
|  |  | Aspirin and statins | 2.15(1.51,3.07)＜0.001 | 1.92(1.28,2.89) 0.002 | 2.04(1.36,3.06) 0.001 | 1.27(0.83,1.95) 0.266 |
|  | **≥30** | Aspirin | 1.35(0.95,1.93) 0.093 | 1.43(0.97,2.09) 0.071 | 1.46(0.98,2.17) 0.062 | 1.19(0.79,1.79) 0.396 |
|  |  | Statins | 1.32(0.89,1.95) 0.168 | 1.33(0.88,2.02) 0.173 | 1.28(0.82,2.02) 0.279 | 0.87(0.54,1.40) 0.564 |
|  |  | Aspirin and statins | 3.23(2.22,4.71)＜0.001 | 2.75(1.79,4.24)＜0.001 | 2.83(1.84,4.36)＜0.001 | 1.78(1.12,2.81) 0.014 |
| **Hypertriglyceridemia** | **no** | Aspirin | 1.15(0.87,1.52) 0.336 | 1.20(0.89,1.63) 0.230 | 1.26(0.92,1.71) 0.150 | 1.05(0.73,1.49) 0.801 |
|  |  | Statins | 1.77(1.28,2.44) 0.001 | 1.90(1.36,2.67)＜0.001 | 1.96(1.40,2.76)＜0.001 | 1.41(0.91,2.18) 0.120 |
|  |  | Aspirin and statins | 2.54(1.94,3.33)＜0.001 | 2.58(1.91,3.50)＜0.001 | 2.57(1.89,3.50)＜0.001 | 1.89(1.31,2.74) 0.001 |
|  | **yes** | Aspirin | 1.32(0.87,2.00) 0.199 | 1.20(0.89,1.63) 0.230 | 1.22(0.79,1.87) 0.368 | 1.06(0.66,1.69) 0.815 |
|  |  | Statins | 0.80(0.54,1.19) 0.266 | 1.90(1.36,2.67)＜0.001 | 0.73(0.47,1.13) 0.155 | 0.55(0.32,0.96) 0.036 |
|  |  | Aspirin and statins | 1.22(0.87,1.71) 0.254 | 2.58(1.91,3.50)＜0.001 | 1.14(0.78,1.67) 0.486 | 1.07(0.68,1.70) 0.769 |
| **Diabetes** | **no** | Aspirin | 0.98(0.74,1.29) 0.874 | 0.99(0.73,1.34) 0.944 | 1.04(0.77,1.40) 0.815 | 1.05(0.75,1.48) 0.772 |
|  |  | Statins | 1.42(1.03,1.96) 0.032 | 1.49(1.07,2.08) 0.019 | 1.47(1.04,2.06) 0.028 | 1.28(0.80,2.06) 0.299 |
|  |  | Aspirin and statins | 1.74(1.31,2.32)＜0.001 | 1.69(1.23,2.31) 0.001 | 1.73(1.26,2.39) 0.001 | 1.56(1.08,2.24) 0.017 |
|  | **yes** | Aspirin | 1.09(0.70,1.68) 0.712 | 1.16(0.73,1.83) 0.535 | 1.04(0.65,1.67) 0.869 | 0.95(0.58,1.56) 0.833 |
|  |  | Statins | 0.68(0.44,1.05) 0.082 | 0.67(0.42,1.07) 0.092 | 0.63(0.40,1.02) 0.059 | 0.54(0.31,0.93) 0.028 |
|  |  | Aspirin and statins | 1.24(0.85,1.81) 0.269 | 1.42(0.93,2.17) 0.108 | 1.43(0.94,2.18) 0.093 | 1.37(0.86,2.16) 0.181 |

**Note:** Model 1: Non-adjusted model; Model 2 adjusted for: gender; age; race; Model 3 adjusted for: education, poverty income ratio, smoking status, alcohol consumption; HBV infection; HCV infection; alanine aminotransferase, and physical activity status in addition to model 2. Model 4: BMI, diabetes, hypertriglyceridemia in addition to model 3.

**Abbreviations:** MASLD, metabolic dysfunction-associated steatotic liver disease; OR, odds ratio; 95%CI, 95% confidence interval.

**Supplementary table 3 |** Subgroup and interaction analyses for the impact of covariates on the association of aspirin use and statins use with the prevalent of advanced liver fibrosis.

|  |  |  | **Model1 OR (95%CI), *P*** | **Model2 OR (95%CI), *P*** | **Model3 OR (95%CI), *P*** | **Model4 OR (95%CI), *P*** |
| --- | --- | --- | --- | --- | --- | --- |
| **Advanced LF** |  |  |  |  |  |  |
| **Gender** | **Male** | Aspirin | 2.20(1.37,3.53) 0.001 | 1.46(0.89,2.41) 0.133 | 1.43(0.87,2.35) 0.157 | 1.08(0.64,1.84) 0.770 |
|  |  | Statins | 3.32(2.04,5.41)＜0.001 | 2.24(1.33,3.76) 0.002 | 2.34(1.33,4.10) 0.003 | 1.58(0.87,2.85) 0.131 |
|  |  | Aspirin and statins | 4.22(2.82,6.30)＜0.001 | 2.38(1.58,3.59)＜0.001 | 2.66(1.76,4.02)＜0.001 | 1.45(0.92,2.27) 0.109 |
|  | **Female** | Aspirin | 2.27(1.51,3.40)＜0.001 | 1.51(0.98,2.33) 0.063 | 1.53(0.98,2.37) 0.059 | 1.15(0.72,1.84) 0.558 |
|  |  | Statins | 2.63(1.71,4.03)＜0.001 | 1.77(1.11,2.83) 0.017 | 1.70(1.05,2.75) 0.031 | 1.13(0.69,1.86) 0.632 |
|  |  | Aspirin and statins | 3.97(2.75,5.73)＜0.001 | 2.25(1.51,3.35)＜0.001 | 2.13(1.42,3.19)＜0.001 | 1.41(0.90,2.22) 0.137 |
| **Age (years)** | **40~59** | Aspirin | 1.87(0.98,3.58) 0.057 | 1.83(0.95,3.53) 0.072 | 1.61(0.82,3.14) 0.168 | 0.93(0.40,2.12) 0.856 |
|  |  | Statins | 2.98(1.55,5.71) 0.001 | 3.04(1.58,5.88) 0.001 | 3.57(1.81,7.04)＜0.001 | 1.58(0.76,3.29) 0.222 |
|  |  | Aspirin and statins | 1.93(1.05,3.55) 0.033 | 1.94(1.02,3.69) 0.042 | 2.03(1.02,4.04) 0.043 | 0.71(0.32,1.55) 0.388 |
|  | **60~80** | Aspirin | 1.33(0.92,1.91) 0.125 | 1.35(0.93,1.94) 0.110 | 1.37(0.95,1.97) 0.092 | 1.12(0.77,1.63) 0.556 |
|  |  | Statins | 1.61(1.11,2.35) 0.013 | 1.65(1.13,2.41) 0.009 | 1.67(1.15,2.44) 0.008 | 1.28(0.85,1.92) 0.232 |
|  |  | Aspirin and statins | 2.27(1.66,3.10)＜0.001 | 2.25(1.64,3.08)＜0.001 | 2.25(1.64,3.08)＜0.001 | 1.52(1.08,2.14) 0.018 |
| **Race** | **Mexican American** | Aspirin | 2.08(1.00,4.33) 0.051 | 1.46(0.68,3.12) 0.336 | 1.69(0.74,3.83) 0.213 | 1.40(0.59,3.32) 0.450 |
|  |  | Statins | 4.53(2.22,9.22)＜0.001 | 3.48(1.58,7.63) 0.002 | 4.52(1.92,10.67) 0.001 | 2.68(1.13,6.36) 0.026 |
|  |  | Aspirin and statins | 5.58(3.02,10.34)＜0.001 | 3.15(1.72,5.78)＜0.001 | 3.50(1.76,6.97)＜0.001 | 1.62(0.75,3.49) 0.220 |
|  | **Other Hispanic** | Aspirin | 2.17(1.00,4.70) 0.050 | 1.58(0.74,3.36) 0.240 | 1.71(0.78,3.76) 0.182 | 1.04(0.45,2.43) 0.920 |
|  |  | Statins | 3.90(1.79,8.51) 0.001 | 2.80(1.20,6.53) 0.017 | 2.69(1.18,6.14) 0.019 | 1.21(0.50,2.90) 0.671 |
|  |  | Aspirin and statins | 4.88(2.57,9.27)＜0.001 | 2.64(1.33,5.23)0.005 | 2.39(1.10,5.17) 0.028 | 1.04(0.43,2.50) 0.925 |
|  | **Non-Hispanic White** | Aspirin | 2.26(1.48,3.44)＜0.001 | 1.49(0.96,2.32)0.074 | 1.47(0.95,2.29) 0.087 | 1.14(0.71,1.83) 0.580 |
|  |  | Statins | 3.01(1.94,4.65)＜0.001 | 1.97(1.23,3.13)0.004 | 2.03(1.25,3.31) 0.004 | 1.40(0.84,2.34) 0.201 |
|  |  | Aspirin and statins | 3.98(2.75,5.75)＜0.001 | 2.22(1.51,3.25)＜0.001 | 2.20(1.48,3.26)＜0.001 | 1.35(0.86,2.10) 0.190 |
|  | **Non-Hispanic Black** | Aspirin | 1.94(1.26,2.99) 0.003 | 1.46(0.94,2.28) 0.094 | 1.39(0.88,2.21) 0.159 | 1.09(0.64,1.85) 0.762 |
|  |  | Statins | 2.04(1.26,3.32) 0.004 | 1.46(0.89,2.39) 0.138 | 1.38(0.81,2.34) 0.238 | 0.93(0.53,1.62) 0.791 |
|  |  | Aspirin and statins | 3.24(2.17,4.84)＜0.001 | 1.97(1.28,3.05) 0.002 | 2.10(1.35,3.27) 0.001 | 1.42(0.89,2.28) 0.146 |
|  | **Other Races1** | Aspirin | 2.30(0.86,6.12) 0.096 | 1.06(0.38,3.00) 0.910 | 1.20(0.43,3.38) 0.730 | 1.14(0.41,3.19) 0.799 |
|  |  | Statins | 2.94(1.28,6.74) 0.011 | 1.46(0.63,3.40) 0.377 | 1.61(0.68,3.82) 0.282 | 1.28(0.46,3.58) 0.639 |
|  |  | Aspirin and statins | 7.90(3.29,18.96)＜0.001 | 3.04(1.27,7.29) 0.012 | 4.39(1.94,9.93)＜0.001 | 2.03(0.75,5.47) 0.160 |
| **BMI group (kg/m**2)** | **20–25** | Aspirin | 2.27(1.04,4.98) 0.040 | 1.28(0.55,2.97) 0.562 | 1.11(0.54,2.28) 0.782 | 1.01(0.50,2.07) 0.973 |
|  |  | Statins | 2.42(1.05,5.60) 0.039 | 1.46(0.61,3.50) 0.394 | 1.79(0.78,4.14) 0.170 | 1.68(0.67,4.24) 0.269 |
|  |  | Aspirin and statins | 2.44(1.14,5.21) 0.021 | 1.39(0.67,2.90) 0.373 | 1.52(0.72,3.21) 0.274 | 1.21(0.55,2.66) 0.640 |
|  | **25–30** | Aspirin | 2.36(1.23,4.53) 0.009 | 1.36(0.67,2.76) 0.388 | 1.44(0.66,3.13) 0.353 | 1.29(0.58,2.87) 0.531 |
|  |  | Statins | 2.55(1.38,4.74) 0.003 | 1.49(0.79,2.79) 0.217 | 1.50(0.76,2.98) 0.243 | 1.21(0.58,2.50) 0.612 |
|  |  | Aspirin and statins | 5.48(3.25,9.24)＜0.001 | 2.52(1.48,4.29) 0.001 | 2.75(1.60,4.73)＜0.001 | 2.19(1.26,3.81) 0.006 |
|  | **≥30** | Aspirin | 2.22(1.47,3.34)＜0.001 | 1.61(1.05,2.47) 0.028 | 1.56(1.01,2.40) 0.043 | 1.00(0.63,1.60) 0.988 |
|  |  | Statins | 3.07(2.01,4.69)＜0.001 | 2.17(1.36,3.46) 0.001 | 2.15(1.34,3.44) 0.002 | 1.41(0.86,2.29) 0.171 |
|  |  | Aspirin and statins | 3.90(2.73,5.59)＜0.001 | 2.22(1.50,3.29)＜0.001 | 2.19(1.47,3.26)＜0.001 | 1.16(0.74,1.83) 0.519 |
| **Hypertriglyceridemia** | **no** | Aspirin | 2.19(1.53,3.15)＜0.001 | 1.42(0.96,2.09) 0.078 | 1.51(1.03,2.21) 0.033 | 1.24(0.82,1.86) 0.308 |
|  |  | Statins | 3.19(2.14,4.76)＜0.001 | 2.05(1.32,3.19) 0.001 | 2.17(1.35,3.49) 0.001 | 1.42(0.85,2.36) 0.180 |
|  |  | Aspirin and statins | 4.79(3.45,6.66)＜0.001 | 2.52(1.76,3.62)＜0.001 | 2.66(1.84,3.83)＜0.001 | 1.59(1.07,2.36) 0.021 |
|  | **yes** | Aspirin | 2.25(1.25,4.07) 0.007 | 1.60(0.87,2.94) 0.132 | 1.54(0.83,2.87) 0.175 | 0.96(0.49,1.87) 0.908 |
|  |  | Statins | 2.44(1.39,4.27) 0.002 | 1.71(0.95,3.06) 0.074 | 1.69(0.92,3.09) 0.089 | 1.17(0.65,2.11) 0.592 |
|  |  | Aspirin and statins | 3.19(1.97,5.18)＜0.001 | 1.87(1.15,3.04) 0.011 | 1.91(1.18,3.11) 0.009 | 1.14(0.69,1.91) 0.606 |
| **Diabetes** | **no** | Aspirin | 1.74(1.08,2.80) 0.024 | 1.05(0.63,1.76) 0.846 | 1.17(0.71,1.94) 0.538 | 1.22(0.74,2.02) 0.439 |
|  |  | Statins | 2.41(1.46,3.98) 0.001 | 1.41(0.85,2.35) 0.189 | 1.46(0.81,2.64) 0.207 | 1.51(0.82,2.79) 0.184 |
|  |  | Aspirin and statins | 3.14(2.02,4.89)＜0.001 | 1.52(0.97,2.38) 0.070 | 1.72(1.09,2.71) 0.019 | 1.88(1.18,3.01) 0.008 |
|  | **yes** | Aspirin | 1.42(0.89,2.27) 0.137 | 1.06(0.65,1.72) 0.815 | 1.02(0.63,1.67) 0.923 | 0.97(0.58,1.62) 0.913 |
|  |  | Statins | 1.30(0.82,2.07) 0.260 | 1.08(0.65,1.78) 0.768 | 1.07(0.65,1.76) 0.785 | 1.09(0.66,1.78) 0.744 |
|  |  | Aspirin and statins | 1.67(1.14,2.46) 0.009 | 1.14(0.76,1.72) 0.533 | 1.14(0.76,1.71) 0.534 | 1.09(0.72,1.66) 0.693 |

**Note:** Model 1: Non-adjusted model; Model 2 adjusted for: gender; age; race; Model 3 adjusted for: education, poverty income ratio, smoking status, alcohol consumption; HBV infection; HCV infection; alanine aminotransferase, and physical activity status in addition to model 2. Model 4: BMI, diabetes, hypertriglyceridemia in addition to model 3.

**Abbreviations:** Advanced LF: advanced liver fibrosis; OR, odds ratio; 95%CI, 95% confidence interval.
